# Pharmacological inhibition of the PERK pathway modulates hepatocellular carcinoma growth and immune signaling

**DOI:** 10.1002/2211-5463.70252

**Published:** 2026-04-21

**Authors:** Ada Lerma‐Clavero, Maria Kopsida, Nathalie Arendt, Hans Lennernäs, Markus Sjöblom, Femke Heindryckx

**Affiliations:** ^1^ Department of Medical Cell Biology Uppsala University Uppsala Sweden; ^2^ Department of Pharmaceutical Biosciences Uppsala University Uppsala Sweden

**Keywords:** AMG PERK44, carcinogenesis, ER stress, fibrosis, hepatocellular carcinoma, idarubicin, inflammation, PERK

## Abstract

The unfolded protein response (UPR) plays an important role in tumor progression and cellular stress adaptation. In hepatocellular carcinoma (HCC), pharmacological inhibition of the protein kinase R‐like endoplasmic reticulum kinase (PERK) is a potential therapeutic strategy, yet its effects on tumor growth and the microenvironment remain unclear. We investigated the selective PERK inhibitor AMG PERK 44 in a diethylnitrosamine (DEN)‐induced mouse model of advanced HCC. Tumor burden, proliferation, fibrosis, immune‐related gene expression, and ER stress signaling were assessed alongside analyses of single‐cell RNA‐sequencing data from HCC mouse models and liver‐specific PERK knockout mice. Our results show that AMG PERK 44 did not alter tumor number nor cause a decrease in tumor area and proliferation. Furthermore, fibrotic burden was unchanged, although fibrosis architecture and stromal gene expression (TGF‐*β*, CTGF, F4/80) were modified. Despite PERK inhibition, the expression of ER stress associated genes (CHOP, EIF2AK3, ERdj4) increased. Single‐cell analysis revealed context‐dependent PERK activity, highest in dendritic cells and macrophages under inflammatory and tumor conditions, while PERK knockout livers showed impaired UPR responses after tunicamycin treatment. Finally, AMG PERK 44 did not enhance idarubicin efficacy and caused no major off‐target effects. These findings highlight the context‐dependent role of PERK in the HCC microenvironment and its implications for targeting UPR pathways in liver cancer.

**Impact statement**

This study provides an evaluation of PERK as a therapeutic target in hepatocellular carcinoma by demonstrating that its inhibition does not produce the anticipated anti‐tumor effects in advanced disease, but instead exerts nuanced, context‐dependent influences on the tumor microenvironment.

## Graphical abstract

Pharmacological inhibition of PERK in a DEN‐induced mouse model of liver cancer does not reduce tumor burden but alters cellular stress signaling. Despite blocking PERK activity, downstream stress responses, including CHOP expression, remain active, suggesting compensatory mechanisms within the unfolded protein response that may influence tumor behavior and therapeutic outcomes.

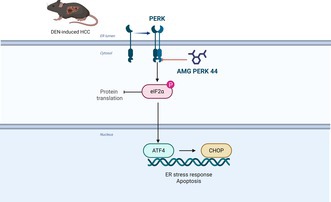

**Abbreviations**
ATF4: Activating transcription factor 4
ATP: Adenosine triphosphate
BSA: Bovine serum albumin
cDNA: Complementary DNA
CHOP (DDIT3): C/EBP homologous protein
CO₂: Carbon dioxide
CTGF: Connective tissue growth factor
DAB: 3,3′‐Diaminobenzidine
DEN: Diethylnitrosamine
DMEM: Dulbecco's modified Eagle medium
DMSO: Dimethyl sulfoxide
ECL: Enhanced chemiluminescence
EIF2AK3 (PERK): Eukaryotic translation initiation factor 2‐alpha kinase 3
ER: Endoplasmic reticulum
ERdj4: Endoplasmic reticulum DnaJ homolog 4
F4/80: Macrophage marker (Adgre1)
GADD34 (PPP1R15A): Growth arrest and DNA damage‐inducible protein 34
GAPDH: Glyceraldehyde 3‐phosphate dehydrogenase
GCN2 (EIF2AK2): General control nonderepressible 2 kinase
GEO: Gene Expression Omnibus
GI: Gastrointestinal
HCC: Hepatocellular carcinoma
H&E: Hematoxylin and eosin
HERP: Homocysteine‐inducible ER protein
HRP: Horseradish peroxidase
IDA: Idarubicin
i.p.: Intraperitoneal
i.v.: Intravenous
KO: Knockout
MASH: Metabolic dysfunction‐associated steatohepatitis
mRNA: Messenger RNA
NC: Normal control
NK cells: Natural killer cells
PBS: Phosphate‐buffered saline
PCA: Principal component analysis
PCNA: Proliferating cell nuclear antigen
PERK: Protein kinase R–like endoplasmic reticulum kinase
pPERK: Phosphorylated PERK
PVDF: Polyvinylidene fluoride
qPCR: Quantitative polymerase chain reaction
RIPA buffer: Radioimmunoprecipitation assay buffer
RMA: Robust Multi‐array Average
ROI: Region of interest
SD: Standard deviation
SR (Sirius Red): collagen staining dye
TGF‐*β*
: Transforming growth factor beta
TNF‐*α*
: Tumor necrosis factor alpha
UMAP: Uniform Manifold Approximation and Projection
UPR: Unfolded protein response
WT: Wild‐type
*α*‐SMA: Alpha‐smooth muscle actin

Hepatocellular carcinoma (HCC) is the most common form of primary liver cancer and a leading cause of cancer‐related mortality worldwide [1, 2]. Its development is often preceded by chronic liver injury, fibrosis, and inflammation, driven by conditions such as viral hepatitis, alcoholism, aflatoxins, and metabolic dysfunction‐associated steatohepatitis [1].

The transition from chronic liver disease to HCC is characterized by genetic and epigenetic alterations in hepatocytes but also by profound remodeling of the liver microenvironment, including fibrosis, immune infiltration, and vascular changes [3, 4, 5]. Despite advances in systemic therapies, treatment options for intermediate‐stage HCC remain limited, and development of resistance to therapy is common [2]. There is an obvious clinical need to identify new therapeutic targets that modulate both tumor cells and their surrounding stroma.

The unfolded protein response (UPR) is a conserved cellular stress response that is activated upon accumulation of misfolded proteins in the endoplasmic reticulum (ER) [6, 7]. One of its sensors is the protein kinase R‐like endoplasmic reticulum kinase (PERK), which is encoded by eukaryotic translation initiation factor 2‐alpha kinase 3 (*EIF2AK3*). Upon activation, PERK phosphorylates eIF2*α* to slow down global protein synthesis, while concurrently promoting the selective translation of transcription factors such as ATF4 and its downstream effectors, including CHOP (*DDIT3*) and GADD34 (*PPP1R15A*). While PERK signaling is essential for maintaining protein homeostasis, prolonged activation can lead to apoptosis. In cancer, PERK has been implicated in promoting tumor growth, survival under hypoxic or metabolic stress, and immune evasion [8, 9]. However, there have been inconclusive reports on its role in HCC, and the effects of PERK inhibition on the fibrotic and immune microenvironment are less understood. Furthermore, it remains unclear whether PERK activity is uniformly required across all liver cell types or selectively activated in specific stromal or immune populations [10, 11].

In this study, we investigated the impact of pharmacological PERK inhibition using AMG PERK 44, a small molecule (molecular mass 561.07 g/mol) that specifically inhibits the PERK pathway [12, 13], in a chemically induced mouse model for HCC [14]. AMG PERK 44 is a recently developed, highly selective small‐molecule inhibitor of the PERK kinase with limited published characterization beyond biochemical and early pharmacodynamic studies [15], and to the best of our knowledge, it has not previously been evaluated in the context of HCC. We assessed its effects on tumor growth, ER stress signaling, fibrosis and immune activation. In parallel, we integrated publicly available transcriptomic and single‐cell RNA‐sequencing datasets to identify how PERK signaling is modulated across immune cell subsets and stress conditions. Furthermore, we combined PERK inhibition with idarubicin (IDA), a commonly used anthracycline in transarterial chemoembolization for HCC treatment, to assess potential synergistic effects [16]. Our results uncover a context‐dependent role for PERK in regulating tumor proliferation, immune signaling, and ER stress adaptation during hepatocarcinogenesis.

## Materials and methods

### Chemicals

Chemicals used during experiments included diethylnitrosamine (DEN; 1 002 877 809, Sigma‐Aldrich, diluted in saline), AMG PERK 44 (SML3049; Sigma‐Aldrich, diluted in saline for *in vivo* or DMSO for *in vitro*), and idarubicin (Zavedos®; Pfizer; Vnr 08 08 20; diluted in DMSO for *in vitro*).

### Animal model

A chemically induced murine model of HCC was used as previously reported [17]. Specifically, 5‐week‐old male sv129 mice (129S2/SvPasCr) underwent intraperitoneal (i.p.) DEN injections (35 mg/kg body weight) biweekly for 28 weeks. The experimental unit was defined as an individual animal. Mice were allocated into the following groups: Healthy, DEN, DEN + AMG PERK 44, and combination treatment DEN + idarubicin + AMG PERK 44 (*n* = 10 per group). Animals were randomly allocated to treatment groups. No predefined exclusion criteria were applied. All animals completing the study were included in the analysis. Beginning at week 25, following tumor formation, mice were treated twice per week with idarubicin intravenous (i.v.) injections (0.4 μg/g bodyweight) and/or AMG PERK 44 i.p. injections (10 μg/g bodyweight). AMG PERK 44 dose was chosen based on prior literature demonstrating *in vivo* efficacy and target engagement [18, 19]. Control group was injected i.v. with saline. After 28 weeks, mice were euthanized by isoflurane anesthesia, while blood was obtained from the ophthalmic venous sinus. After cervical dislocation, relevant tissues were collected for subsequent analyses. Body weight was monitored throughout the study during each injection and at study end‐point. If mice suffered from weight loss ≥ 15% compared to the previous week, an injection was omitted. Humane endpoints were decided prior to the experiment and evaluated using the Uppsala University standards for rodent health. At termination, liver macroscopic tumors were counted, and distal organs were examined for potential metastases. All procedures complied with Uppsala University's ethical guidelines for animal experimentation (DNR 5.8.180089/2020) and adhered to RESIST recommendations. All animals were allowed to acclimatize for at least one week in the Animal Department, prior to the start of the experiment and allowed water and food *ad libitum*. Housing conditions were individually ventilated cages, 21–22 °C at a 12–12 h light–dark cycle with 60% relative humidity. Note that the ‘Healthy’ and ‘DEN’ and ‘DEN + idarubicin’ control groups have also been included in the following publication Lerma‐Clavero A *et al*, 2025 [17], as the animal experiments for both studies were conducted simultaneously, with different treatment arms but shared controls. This dual use of control groups adheres to the 3R principle by minimizing animal use.

### Cell culture and *in vitro* treatments

The human liver cancer cell lines used were hepatoblastoma‐derived HepG2 (ATCC. HB‐8065™) and HCC‐derived Huh7 (kindly gifted from Mårten Fryknäs, Uppsala University, Sweden), along with the hepatic stellate cell line LX‐2 (SCC064; Sigma‐Aldrich, Darmstadt, Germany). All three cell lines were routinely cultured in DMEM, high glucose, GlutaMAX™ Supplement, pyruvate (10 569 010; Thermo Fisher, Uppsala, Sweden), supplemented with 1% penicillin/streptomycin (15 140 122; Thermo Fisher) and 10% fetal bovine serum (10 270 106; Thermo Fisher). Starvation medium (DMEM) was only supplemented with 1% penicillin/streptomycin. Culture medium was replaced every 2–3 days and cells were split at about 70–90% confluency. Cells were maintained in an incubator at 37 °C, 5% CO_2_ and 21% O_2_. Cell lines were authenticated and regularly tested for mycoplasma contamination using Mycostrips.

To evaluate the inhibitory effect of AMG PERK 44 *in vitro*, Huh7 cells (2,5 × 10^5^ cells/well) were seeded at 70–80% confluency in 6‐well plates and allowed to attach for 8 h before changing to starvation media for overnight incubation. Drug treatment was carried out by replacing the starvation medium with fresh complete medium containing DMSO (control) or AMG PERK 44 treatment (0,5 μm or 10 μm) and incubating cells for 6, 24 or 36 h. For the 36‐h time point, medium was replaced with fresh drug‐containing medium after 24 h. Treatment concentrations were based on prior work, showing *in vitro* efficacy [13, 20]. Following treatment, adherent cells were washed with PBS, lysed with either 150 μL/well of RIPA buffer with protease inhibitors (89900, 78 430; Thermo Scientific) for western blotting or 350 μL/well of lysis buffer (R6834‐02; Omega Bio‐tek, Stockholm, Sweden) for qPCR.

For the spheroid monoculture assays, single‐cell solutions (2 × 10^3^ cells in 100 μL/well) were seeded in Nunclon™ Sphera™ 96‐Well, Nunclon Sphera‐Treated, U‐Shaped‐Bottom Micro‐plate to generate spheroids (174 925; Thermo Fisher). For the spheroid coculture assays, single‐cell solutions were prepared and a ratio of 4 : 1 of Huh7/HepG2 and LX‐2, respectively, and seeded per well. After the formation of spheroids in the incubator (72 h), fresh starvation medium (100 μL/well) was added and plates were incubated for 4 h at 37 °C. Stock solutions of IDA were prepared in supplemented medium at 2× final concentration and serially diluted 1 : 2 in fresh medium. Next, fresh medium (100 μL) containing drug was added to each well resulting in a total volume of 200 μL and achieving a 1× drug concentration. Spheroids were treated with IDA (0 to 1000 μm) or IDA (0 to 1000 μm) + AMG PERK 44 (10 μm). Treated spheroids were placed in the incubator for 24 h. After 24 h, viability was measured using the CellTiter‐Glo® 3D Cell Viability ATP‐based assay (G9683; Promega, Nacka, Sweden), following the manufacturer's instructions. Luminescence was recorded with the FLUOstar Omega plate reader.

### Antibodies

Primary antibodies used for immunohistochemistry included Ki67 (ab16667; Abcam, Cambridge, UK; 1 : 200), PCNA (PA5‐27214; Thermo Fisher, 1 : 100), and *α*‐SMA (Abcam: ab5694; 1 : 100). Primary antibodies used for immunofluorescence were ATF4 (PA5‐27576; Invitrogen, Stockholm, Sweden; 1 : 100), F4/80 [CI:A3‐1] (ab6640; Abcam, 1 : 100). Secondary antibodies used for immunofluorescence included donkey anti‐rabbit Alexa Fluor™ 488 (A‐21206; Invitrogen, 1 : 500) and donkey anti‐goat Alexa Fluor™ 555 (A‐21432; Invitrogen, 1 : 500). Primary antibodies used for western blotting were ATF4 (PA5‐27576; Invitrogen, 1 : 500), p PERK/EIF2AK3 (Thr982) (82534‐1‐RR; Proteintech, Manchester, UK; 1 : 2000), Anti‐PERK [EPR19876‐294] (ab229912; Abcam, 1 : 1000), and GAPDH (4A9L6) (MA5‐35235; Thermo Fisher, 1 : 50000). Secondary antibody used for western blotting was goat anti‐rabbit IgG H&L (HRP) (ab6721; Abcam, 1 : 3000).

### Histological staining and immunohistochemistry

Tissues were fixed in 4% paraformaldehyde for 24 h and then processed for paraffin embedding. Paraffin blocks were sectioned at a thickness of 5 μm using a microtome, and the sections were air‐dried overnight. After deparaffinization and rehydration, tissue sections were subjected to hematoxylin and eosin (H&E), Sirius Red (SR), Proliferating Cell Nuclear Antigen (PCNA), or Alcian Blue‐PAS (pH 2.5; 60122.09, Thermo Fisher) stainings following established protocols. Immunohistochemical analyses were carried out with a rabbit‐specific HRP/DAB detection IHC kit (ab64261; Abcam) in accordance with the manufacturer's recommendations. Antigen retrieval was performed by heating the slides in a decloaking chamber containing DIVA decloaker 10× (DV2004MX; Biocare Medical, Gothenburg, Sweden) for 1.5 h. Primary antibody incubation was conducted for 1 h at 37 °C. Stained sections were imaged using an Axio Scope microscope (Carl Zeiss, Stockholm, Sweden; 10×/0.25 objective), and image acquisition and processing were performed with ZEN 3.2 microscopy software (ZEISS, Stockholm, Sweden).

### Immunofluorescence

Paraffin‐embedded tissue blocks were sectioned at 5 μm thickness, followed by deparaffinization and rehydration. Antigen retrieval was carried out by incubating the sections for 1.5 h in a decloaking chamber using DIVA Decloaker 10× (DV2004MX; Biocare Medical). The sections were subsequently blocked for 1 h with 1% BSA in PBS containing Tween‐20 and incubated overnight with primary antibodies diluted in the blocking buffer. The following day, sections were treated with secondary antibodies in blocking buffer for 45 min at room temperature, protected from light. Nuclear staining was performed using NucBlue™ (Hoechst 33342; R37605; Invitrogen) at a dilution of 1 : 1000. Fluorescent images were acquired with a slide scanner (Zeiss Axio Scan.Z1) using a Plan Apochromat (20×/0,8) M27 Zeiss objective.

### Image analysis

Image analysis was performed using fiji/imagej software. For H&E‐stained sections, tumor regions were identified, and the corresponding tumor areas were quantified. Sirius Red‐stained sections were evaluated according to the METAVIR scoring system. Quantification of collagen deposition was performed in a blinded manner by converting images to grayscale, followed by automated detection of stained regions on thresholded images using a customized macro. For *α*‐SMA staining, a median filter with a radius of 4 was first applied, after which color deconvolution was used to isolate the DAB signal [21]. The resulting DAB image was thresholded, and regions of interest (ROIs) were measured. Ki67 staining was analyzed following a similar procedure, using a median filter with a radius of 3; particles within a predefined size range (≥ 70 μm^2^) were quantified after thresholding. Fluorescent images were analyzed by separating individual channels, followed by quantification of the positive signal area after thresholding. For each slide (one slide per mouse), 10 images from randomly selected areas were acquired and analyzed. Automatic thresholding (default) was applied to the NucBlue channel to detect nuclei. For the ATF4 channel, the threshold was manually adjusted to exclude background and accurately segment the positive area. Lastly, ATF4 and F4/80 signals were normalized to the NucBlue‐positive area to account for differences in cell number. Image analysis of intestinal samples was performed using method‐specific approaches. For H&E‐stained sections, villus height, villus width, and crypt depth were measured using straight‐line tools at the widest points from three representative sections per sample, as previously described [22, 23]. For PCNA staining, the positively stained area was quantified through color deconvolution and image thresholding (particle size range: 0–100), and results were expressed as the ratio of positive staining area to total tissue area. Alcian Blue‐stained samples were analyzed in a similar manner, except that each positively stained goblet cell was manually counted.

### Quantitative RT‐PCR of mRNA

Total RNA was isolated using the E.Z.N.A. Total RNA Kit I (R6834‐02; Omega Bio‐tek) according to the manufacturer's instructions. RNA concentration and purity were determined using a NanoDrop spectrophotometer. Complementary DNA (cDNA) was synthesized from total RNA using the iScript cDNA Synthesis Kit (1 708 891; Bio‐Rad, Solna, Sweden) following the manufacturer's protocol.

Gene‐specific primers were designed using NCBI Primer‐BLAST and synthesized by Thermo Fisher Scientific. Quantitative PCR was performed in duplicate in a 384‐well plate using Fast SYBR Green Master Mix (434 385 612; Thermo Fisher Scientific) under standard cycling conditions. Amplification and data acquisition were carried out on a QuantStudio 5 Real‐Time PCR System (Thermo Fisher Scientific). Relative gene expression was calculated using the 2‐ΔΔ Ct method. Expression levels of target genes were normalized to the reference genes GAPDH and *β*‐actin, and results were expressed relative to the healthy control group.

### Gene expression analysis of liver‐specific PERK knockout mice

We obtained a publicly available microarray dataset (GSE29929) from the NCBI Gene Expression Omnibus. This dataset comprises liver samples from wild‐type (WT) and liver‐specific PERK knockout (PERK^−/−^) mice, each either untreated (DMSO control) or treated with the ER stress inducer tunicamycin for 6 h [24]. In total, 14 Affymetrix Mouse Genome 430 2.0 arrays were analyzed: 3 WT controls, 3 KO controls, 4 WT treated, and 4 KO treated. Raw expression data (CEL files) were imported and normalized using the affy package in R (v4.3.2). Background correction, normalization, and summarization were performed using the Robust Multi‐array Average (RMA) method. Probe sets were annotated using the mouse4302.db package, and probes without valid gene symbols were excluded. When multiple probes mapped to the same gene, the probe with the highest average expression across samples was retained. To assess expression changes in UPR‐related genes (e.g., *Ddit3*, *Atf4*, *Hspa5*, *Herpud1*, *Ero1lb*, *Edem1*, *Eif2ak3*), and inflammation/fibrosis markers (e.g., *Tnf*, *Il6*, *Il1b*, *Cxcl10*, *Col1a1*, *Tgfb1*, *Acta2*, *Adgre1*, *Timp1*, *Il16*), expression values for each sample were normalized to the average expression of WT control samples (fold change vs WT control). Data were reshaped using reshape2, individual‐level data tables and normalized expression matrices were exported for further visualization in graphpad Prism.

### Single‐cell RNA‐sequencing data from mouse liver immune cells

Single‐cell RNA‐sequencing data from mouse liver immune cells was obtained from the Gene Expression Omnibus (GEO) under accession number GSE282630 [25]. The dataset includes CD45^+^ cells isolated from mice across four conditions: normal control (NC), metabolic dysfunction‐associated steatohepatitis (MASH), nontumor paracancerous tissue (HCC_N), and hepatocellular carcinoma (HCC_T). Raw count matrices (MTX format) were downloaded and imported into R using the Seurat package (v5.0.1). Barcodes, features, and expression matrices were loaded for each sample and converted into Seurat objects. Cells were filtered based on quality metrics if needed, and datasets were merged and normalized using *Seurat's SCTransform* pipeline. Dimensionality reduction was performed using principal component analysis (PCA) followed by Uniform Manifold Approximation and Projection (UMAP). Immune cell identities were predicted using the *SingleR* package with reference to the *MouseRNAseqData* collection from *celldex*. Predicted labels were added to the Seurat metadata for downstream visualization and analysis.

To assess general UPR activation, a UPR score was calculated using the *AddModuleScore* function in Seurat based on curated PERK target genes (*Eif2ak3, Atf4, Ddit3, Ppp1r15a, Hspa5, Asns*, and others). UMAP plots, violin plots, and gene expression overlays were generated using Seurat's built‐in visualization functions and customized with ggplot2. All analyses were performed in R (v4.5.1).

### Western blotting

Samples in RIPA buffer were sonicated for 30 s at 20% amplitude and stored at −20 °C until use. Prior to electrophoresis, protein concentrations were determined using the Pierce™ BCA Protein Assay (23228; Thermo Scientific) and the albumin standard (23209; Thermo Scientific) according to the manufacturer's instructions. Equal amounts of protein (40 μg from animal samples and 11 μg from *in vitro* samples) were loaded on Mini‐PROTEAN TGX gels (4569036, 4 569 034; Bio‐Rad) and ran in 1× Tris Glycine SDS buffer (Bio‐Rad) at 100 V for 60 min. Gels were activated by a 5 min exposure using an Imaging System (ChemiDoc™ Touch, Bio‐Rad). Proteins were then transferred to a Minisize PVDF membrane (Bio‐Rad) using a semi‐dry transfer (TurboBlot, Bio‐Rad) for 30 min using the standard built‐in protocol. Membranes were imaged using the Imaging System (ChemiDoc™ Touch, Bio‐Rad) to obtain images of total protein and subsequently equilibrated in TBS‐T (0.2% Tween‐20 in TBS) for 15 min. To block nonspecific binding, membranes were incubated in TBS‐T containing 5% BSA for 1 h at room temperature. Membranes were then incubated with primary antibodies overnight at 4 °C. The next day, membranes were washed in TBS‐T and incubated for 1 h at room temperature with the secondary antibody. Finally, membranes were washed in TBS‐T and incubated with ECL Clarity Substrate and Reagent (Bio‐Rad: 1705060) for 5 min and imaged for chemiluminescence signal (ChemiDoc™ Touch, Bio‐Rad). After detection, membranes were stripped using a mild stripping buffer (glycine, SDS, Tween‐20 and deionized water; pH 2.2) for 15 min at room temperature, followed by washes with TBS‐T before reprobing with primary antibodies.

### Statistical analysis

Data are expressed as mean ± standard deviation (SD), and differences were considered statistically significant at *P* < 0.05. Statistical analyses were performed using one‐way analysis of variance (ANOVA) followed by Tukey's *post hoc* multiple comparisons test. For cell viability assays, each data point represents the mean of technical replicates from two independent biological experiments, and statistical significance was assessed by nonlinear regression analysis. For qPCR data, outliers were identified and excluded using the ROUT method prior to analysis. Cleaned datasets were analyzed using two‐way ANOVA followed by Tukey's multiple comparisons test. For intestinal samples, statistical analysis was performed using an ANOVA with Tukey's HSD post hoc test, with comparisons of *P* < 0.05 indicating significance. Where applicable, pairwise comparisons were performed with a Student's *t*‐test. All statistical analyses and data visualizations were performed with graphpad Prism version 9 (graphpad Software, San Diego, CA, USA).

## Results

### Effects of AMG PERK 44 treatment on tumor burden and proliferation

**Fig. 1:**
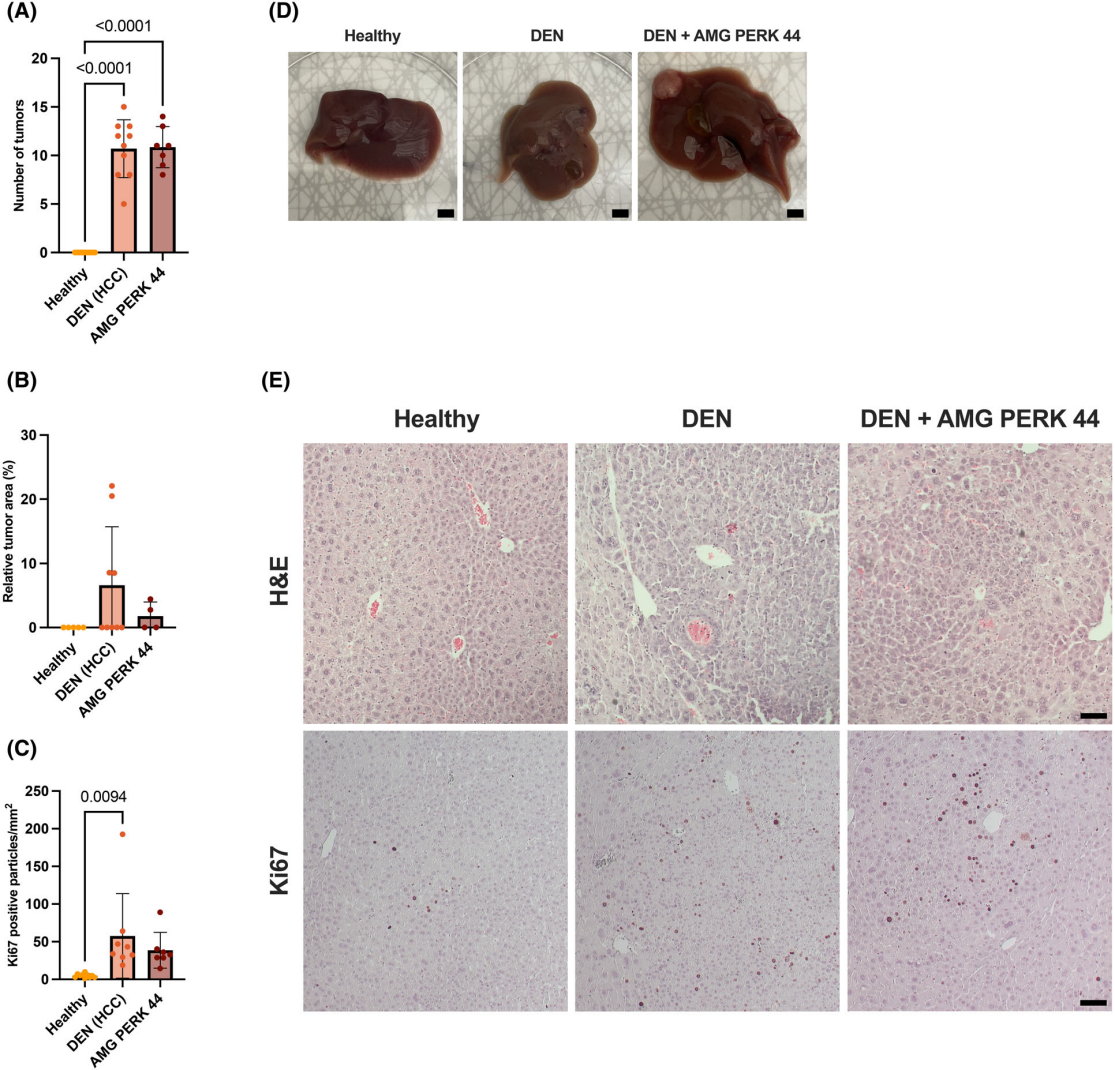
Effects of AMG PERK 44 treatment on tumor burden and proliferation. (A) Quantification of tumor number per liver in healthy, DEN (HCC), and AMG PERK 44‐treated mice. (B) Relative tumor area expressed as a percentage of total liver area. (C) Quantification of Ki67‐positive nuclei per mm^2^ as an index of proliferative activity. (D) Macroscopic images of mice livers. Scale bars represent 100 pixels. (E) Representative images of hematoxylin and eosin (H&E) and Ki67 immunostaining from liver sections of the indicated groups. Each data point represents one mouse. *N* = 7–10 mice per group. All data are expressed as mean ± SD. Statistical significance was determined using one‐way ANOVA followed by Tukey's multiple comparisons test. Scale bars represent 100 μm.

To assess the impact of AMG PERK 44 in the context of HCC, we first evaluated general tumor burden across the three experimental groups: Healthy, DEN‐induced HCC (DEN), and DEN + AMG PERK 44. To determine the impact of PERK inhibition on tumor development and growth, we quantified the number and size of liver tumors, as well as cellular proliferation rates across the DEN and DEN+AMG PERK 44 groups. Both DEN and DEN+AMG PERK 44 mice developed approximately ten macroscopically visible tumors per mouse, with no significant differences in tumor number between the two groups (Fig. 1A). When evaluating the relative tumor area (expressed as a percentage of total liver area), we observed a nonsignificant reduction in AMG PERK 44‐treated mice compared to DEN controls (Fig. 1B). This suggests that while tumor initiation remained unaffected, tumor growth was slightly attenuated following PERK inhibition. To further assess proliferative activity, we quantified Ki67‐positive nuclei in liver sections. As expected, DEN‐induced tumors showed a significant increase in Ki67 staining compared to healthy liver tissue (Fig. 1C). In the DEN+AMG PERK 44 group, Ki67 positivity was slightly reduced compared to DEN controls, although not completely normalized. Together, these findings indicate that AMG PERK 44 given i.p. biweekly during three weeks does not prevent tumor formation but may limit tumor expansion by suppressing cell proliferation.

### Fibrosis and stromal remodeling

**Fig. 2:**
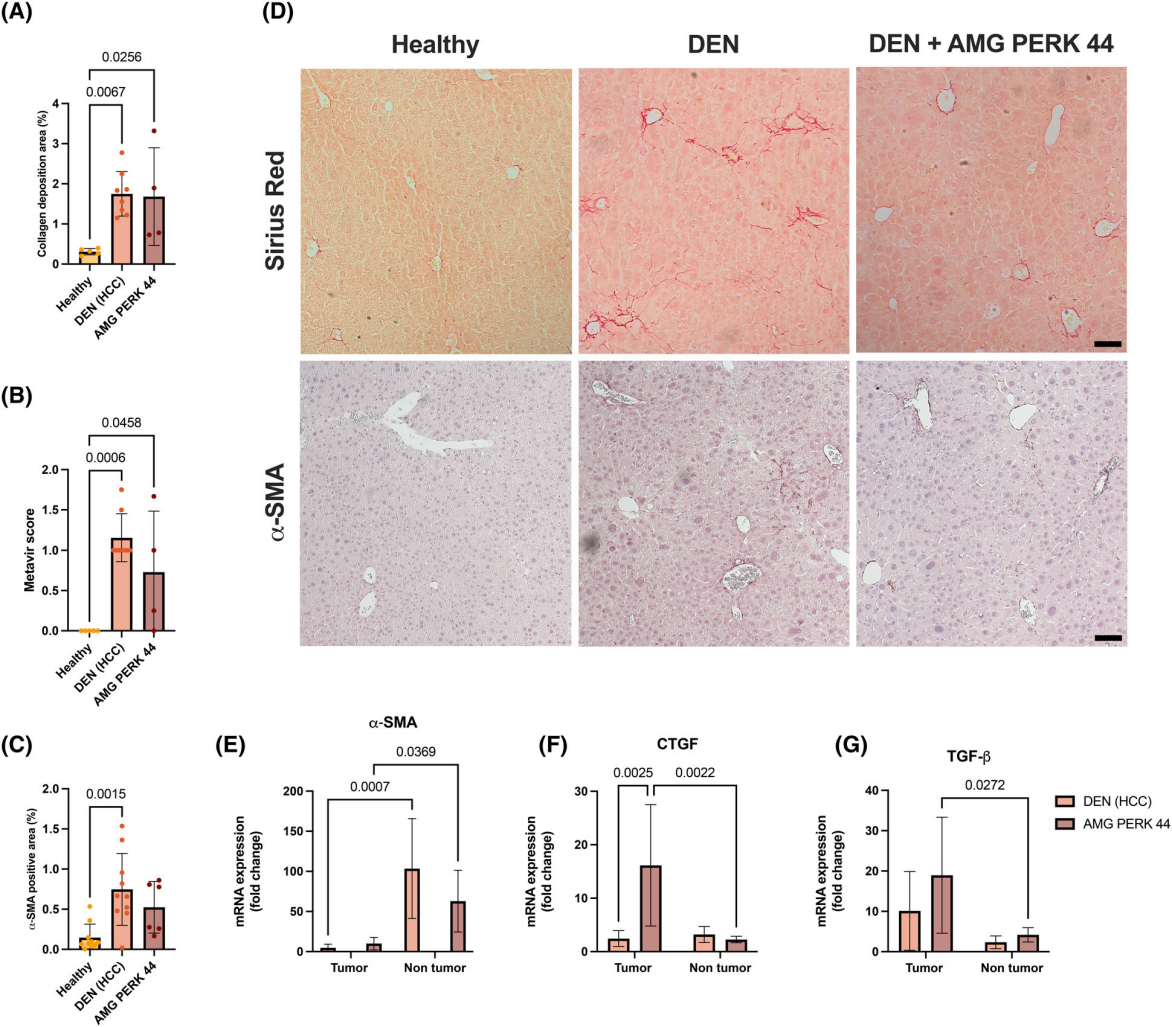
Impact of AMG PERK 44 treatment on fibrosis and inflammation. (A) Quantification of collagen deposition area (%) as assessed by histological analysis of Sirius Red staining. (B) Fibrosis severity determined using the Metavir scoring system. (C) Quantification of *α*‐SMA‐positive area (%) as a marker of hepatic stellate cell activation. (D) Representative images of Sirius Red and *α*‐SMA staining from liver tissue. Statistical significance was determined using one‐way ANOVA followed by Tukey's multiple comparisons test. (E–G) Relative mRNA expression levels of profibrotic genes in tumor and nontumor liver tissue, including *α*‐SMA (E), CTGF (F), and TGF‐*β* (G), as determined by qRT‐PCR. Data are presented as fold change relative to healthy liver controls (set to 1). Relative gene expression was analyzed using two‐way ANOVA followed by Tukey's multiple comparisons test. Data are presented as mean ± SD. Each data point represents one mouse. *N* = 4–10 mice per group. Scale bars represent 100 μm.

**Fig. 3:**
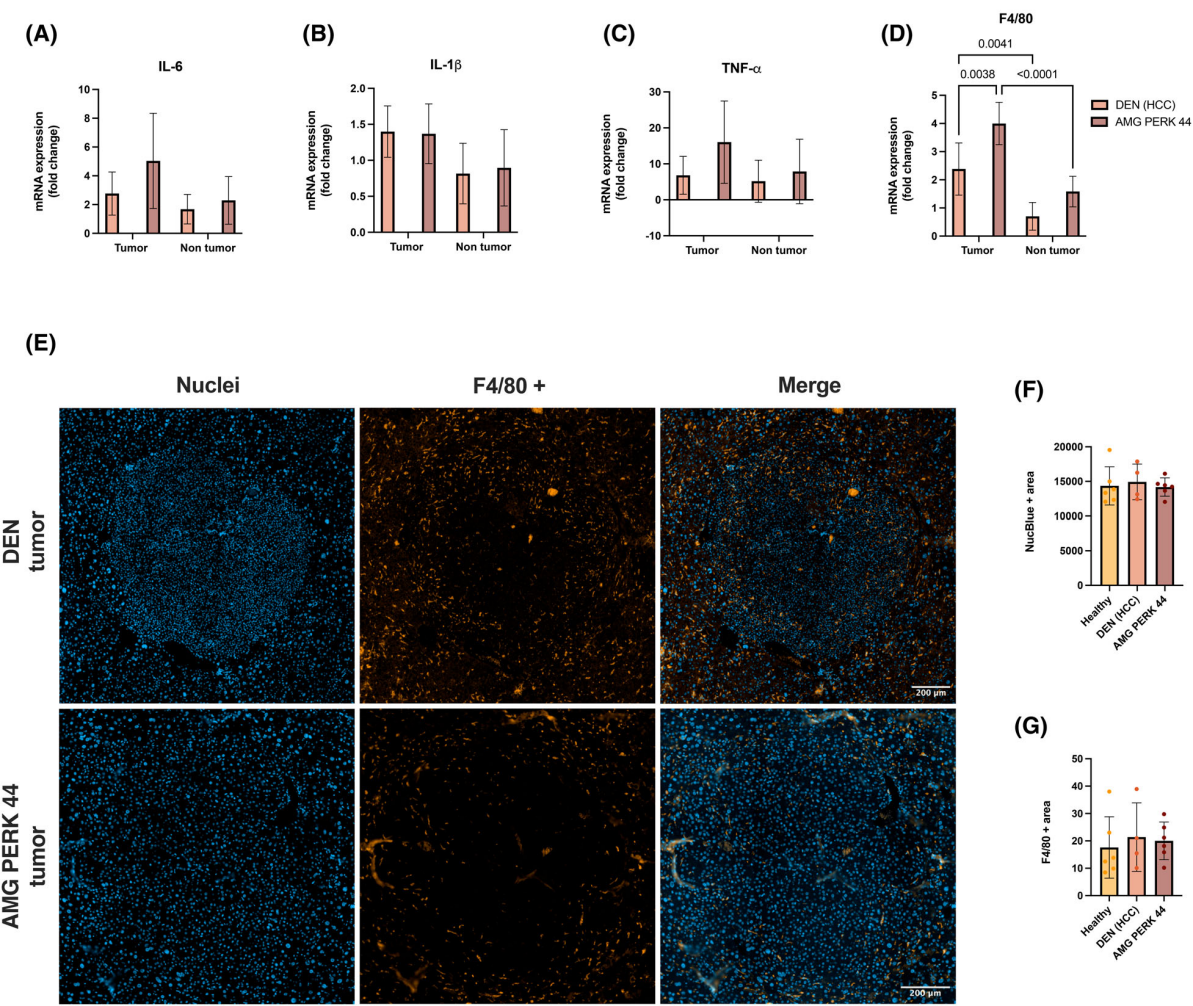
Impact of AMG PERK 44 treatment on inflammation. (A–D) Quantitative RT–PCR analysis of IL‐6, IL‐1*β*, TNF‐*α*, and F4/80 mRNA expression in tumor and nontumor liver tissue. Data are presented as fold change relative to healthy liver controls (set to 1). Relative gene expression was analyzed using two‐way ANOVA followed by Tukey's multiple comparisons test. (E) Representative images of nuclei and F4/80 immunofluorescent staining on tumors from DEN and AMG PERK 44‐treated mice. Scale bars represent 200 μm. (F) Quantification of nuclei (Nucblue‐positive raw area) and (G) F4/80‐positive area (normalized to Nucblue‐positive area). Statistical significance was determined using one‐way ANOVA. Data are presented as mean ± SD. Each data point represents one mouse. *N* = 4–6 mice per group. Scale bars represent 200 μm.

To evaluate whether PERK inhibition affects the fibrotic tumor microenvironment [26, 27], we assessed collagen deposition, fibrosis scoring, myofibroblast activation, and the expression of fibrosis and inflammation‐associated genes in tumor and nontumor regions. Quantification of collagen content by histological staining revealed no difference in the percentage of collagen area between DEN‐induced HCC and AMG PERK 44‐treated mice (Fig. 2A). However, fibrosis patterning, as reflected by the Metavir score, was slightly reduced in the AMG PERK 44 group, but this was not statistically significant (Fig. 2B). Staining for *α*‐smooth muscle actin (*α*‐ SMA), a marker of activated myofibroblasts, showed a clear increase in DEN‐induced HCC compared to healthy controls (Fig. 2C). AMG PERK 44 treatment led to a modest but nonsignificant reduction in aSMA‐positive area. Gene expression analysis further revealed that *α*‐SMA mRNA was significantly higher in the nontumor compartment compared to tumor tissue (Fig. 2E). Conversely, in the tumor compartment, mRNA levels of connective tissue growth factor (CTGF) were significantly increased in AMG PERK 44‐treated mice, while expression in the nontumor compartment decreased (Fig. 2F). TGF‐*β* mRNA expression followed a similar pattern as CTGF but did not reach statistical significance (Fig. 2G). IL‐6 mRNA expression was slightly elevated in tumors from AMG PERK 44‐treated animals, whereas IL‐1B expression remained unchanged across groups and compartments (Fig. 3A,B). TNF‐*α* expression was higher in the tumor and nontumor compartment of AMG PERK 44‐treated mice, although this increase was not statistically significant (Fig. 3C). Assessment of immune cell infiltration showed that F4/80 mRNA, a marker for macrophages, was significantly increased in tumor tissue compared to nontumor tissue in both DEN and AMG PERK44 groups. Moreover, AMG PERK 44 treatment significantly increased F4/80 mRNA levels in tumor tissue (Fig. 3D). Histological examination indicated a consistent pattern in which macrophages accumulated predominantly at the tumor periphery rather than infiltrating deeply into the tumor mass (Fig. 3E). However, quantification of the F4/80‐positive area in tissue sections revealed no significant differences among the groups (Fig. 3F,G). These data suggest that AMG PERK 44 treatment does not reduce overall fibrosis levels at this dose. However, it might alter fibrosis architecture, modulate stromal gene expression, and promote immune‐related signaling in the tumor microenvironment.

### Immune landscape

**Fig. 4:**
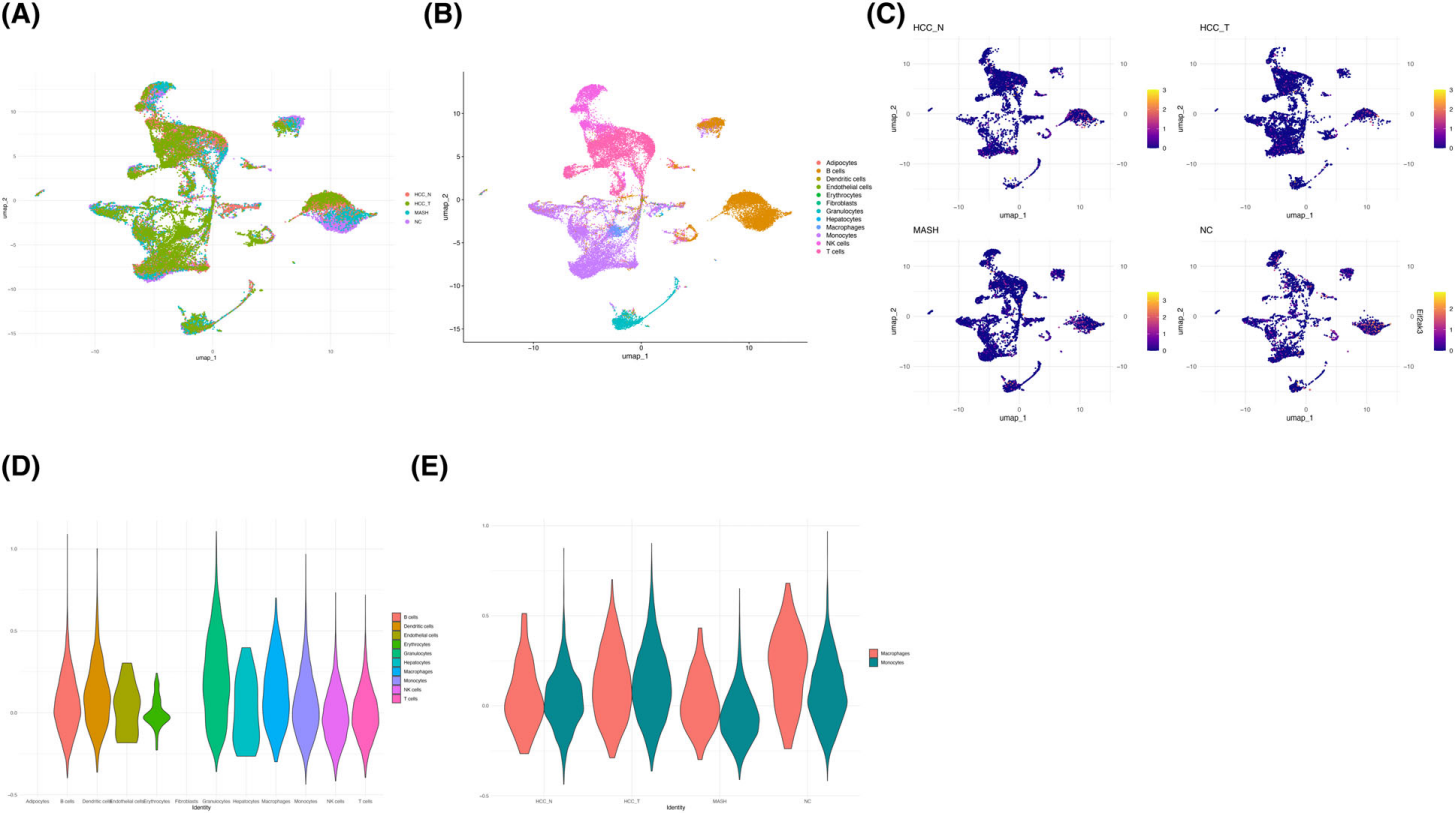
PERK/UPR pathway activity in immune cells across liver disease progression. (A) UMAP projection of CD45^+^ immune cells from mouse livers (GSE282630), color‐coded by condition: normal control (NC), metabolic dysfunction‐associated steatohepatitis (MASH), nontumor tissue adjacent to tumor (HCC_N), and tumor (HCC_T). (B) UMAP projection of immune cell types. (C) UMAP split by condition, displaying expression of Eif2ak3 (PERK). (D) Violin plot showing PERK/UPR module scores across annotated immune cell types, calculated from a curated set of PERK/ATF4 downstream target genes. (E) Violin plot showing PERK/UPR module scores in macrophages and monocytes stratified by condition.

To investigate how immune cells evolve during liver disease progression, we analyzed publicly available single‐cell RNA‐sequencing data (GSE282630) consisting of CD45^+^ immune cells isolated from mouse livers at four disease stages: normal control (NC), metabolic dysfunction‐associated steatohepatitis (MASH), nontumor paracancerous tissue (HCC_N), and hepatocellular carcinoma (HCC_T) (16). UMAP projection revealed a largely conserved immune cell landscape across conditions, with most clusters containing cells from all four groups (Fig. 4A). Nonetheless, some immune cell types, such as monocytes, macrophages, NK cells, and T cells, were more abundant in MASH and HCC, suggesting disease‐specific changes in the immune landscape, as expected (Fig. 4B). Expression of *Eif2ak3*, the gene encoding PERK, was generally low but detectable in discrete immune cell subsets across all conditions (Fig. 4C). A modest increase in *Eif2ak3*‐positive cells was observed in MASH and HCC_T, suggesting that transcriptional upregulation of PERK may occur in response to inflammatory or tumor‐related stress in a subset of immune cells. To gain broader insight into stress‐responsive transcriptional programs associated with PERK signaling, we calculated a UPR‐associated gene module score based on expression levels of canonical downstream targets, including *Atf4*, *Ddit3*, *Ppp1r15a*, and *Asns*. Importantly, these genes are not exclusively regulated by PERK and the score therefore reflects general UPR activation rather than PERK kinase activity specifically. This score varied across immune cell types, with dendritic cells and granulocytes exhibiting the highest UPR activity, followed by macrophages and monocytes (Fig. 4D). In contrast, lymphoid populations such as T cells, NK cells, and B cells displayed consistently low scores. Stratifying macrophages and monocytes by condition revealed that UPR transcriptional activation was high in normal tissue (NC), with a slight elevation in tumor tissue (HCC_T), and decreased in both MASH and HCC_N (Fig. 4E,F). Altogether, these findings indicate that UPR transcriptional activation is most prominent in myeloid‐derived immune cells. Because the score reflects downstream transcriptional outputs rather than PERK phosphorylation or kinase activity, it should be interpreted as a measure of UPR pathway engagement, not direct PERK activation.

### ER stress signaling

**Fig. 5:**
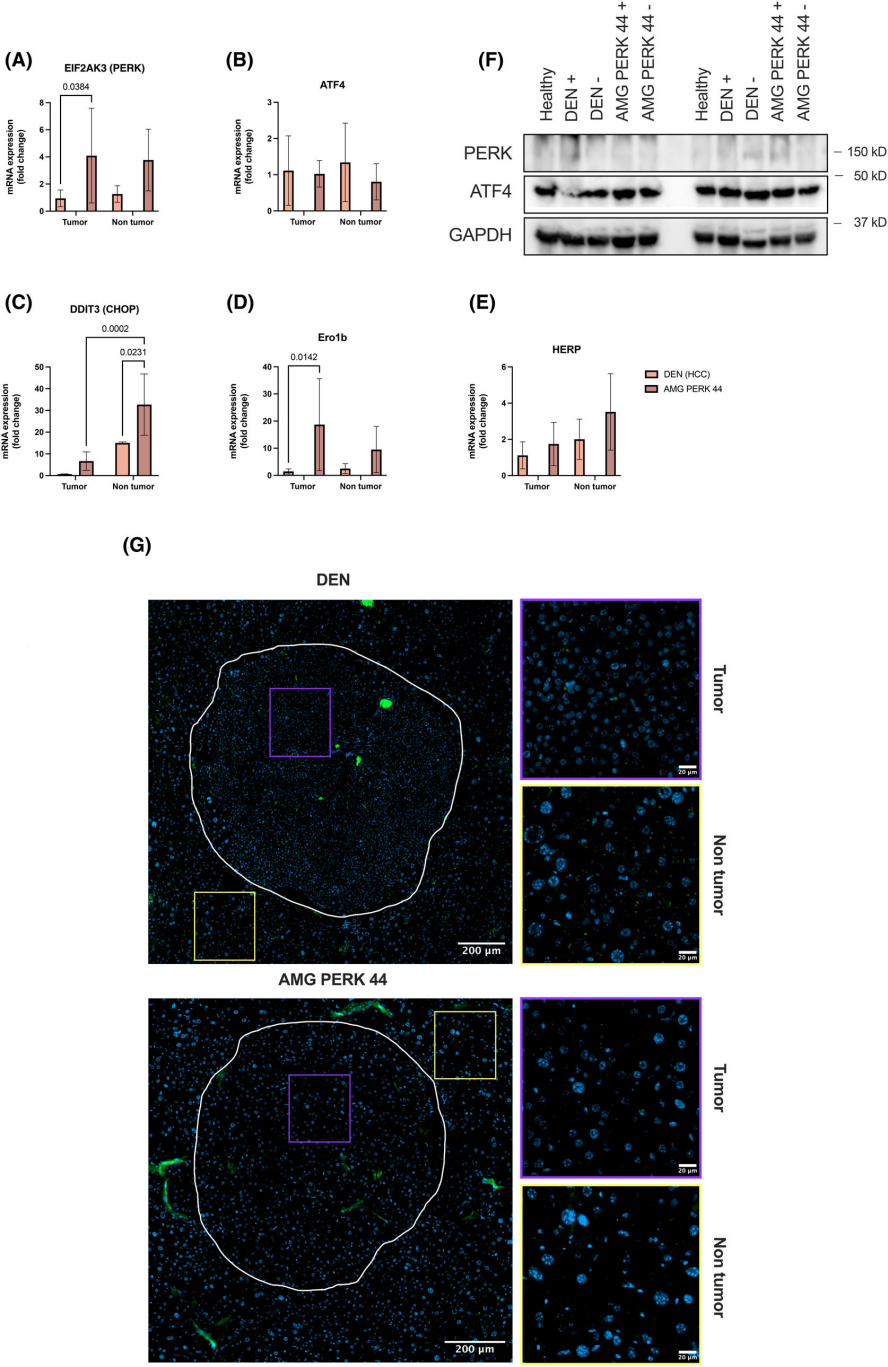
Effect of AMG PERK 44 treatment on UPR markers *in vivo*. mRNA expression levels of EIF2AK3 (PERK) (A), ATF4 (B), DDIT3 (CHOP) (C), Ero1b (D), and HERP (E) were quantified by qRT–PCR in tumor and liver tissue from mice with DEN‐induced hepatocellular carcinoma (HCC) treated with vehicle DEN (HCC) or the PERK inhibitor AMG PERK 44. Data are expressed as fold change relative to healthy liver controls (set to 1). Bars represent mean ± SD. Relative gene expression was analyzed using two‐way ANOVA followed by Tukey's multiple comparisons test. (F) Western blot analysis of PERK and ATF4 in tissue lysates from healthy, DEN and AMG PERK 44‐treated mice. Tumor samples are indicated as ‘+’and nontumor samples as ‘‐’. Each lane represents an independent biological sample from the indicated group. GAPDH was used as a loading control. (G) Representative images of nuclei and F4/80 immunofluorescent staining on tumors from DEN and AMG PERK 44‐treated mice. Scale bars represent 200 μm and 20 μm.

To evaluate the impact of AMG PERK 44 on ER stress signaling *in vivo*, we assessed gene expression and protein markers associated with the PERK pathway in tissue. Contrary to expectations, pharmacological inhibition of PERK at this dose did not lead to a reduction of mRNA expression in the ER stress markers. Instead, mRNA expression of Eif2ak3 (encoding PERK), DDIT3 (encoding CHOP), Ero1b, and HERP was increased in tumor and nontumor tissues from AMG PERK 44‐treated mice compared to untreated DEN‐HCC controls (Fig. 5A–E). This may suggest activation of compensatory or feedback mechanisms within the ER stress pathway despite PERK inhibition. However, PERK protein was not detected by western blot, despite measurable mRNA expression levels (Fig. 5F). Interestingly, mRNA levels of ATF4, a direct downstream target of Eif2ak3/PERK, remained unchanged upon AMG PERK 44 treatment (Fig. 5B). Consistent with the mRNA data, western blot (Fig. 5F) and immunofluorescent staining (Fig. 5G) of ATF4 showed clear ATF4 protein expression, but no differences between conditions. Together, these findings highlight a potential paradoxical upregulation of ER stress‐related transcripts and proteins.

**Fig. 6:**
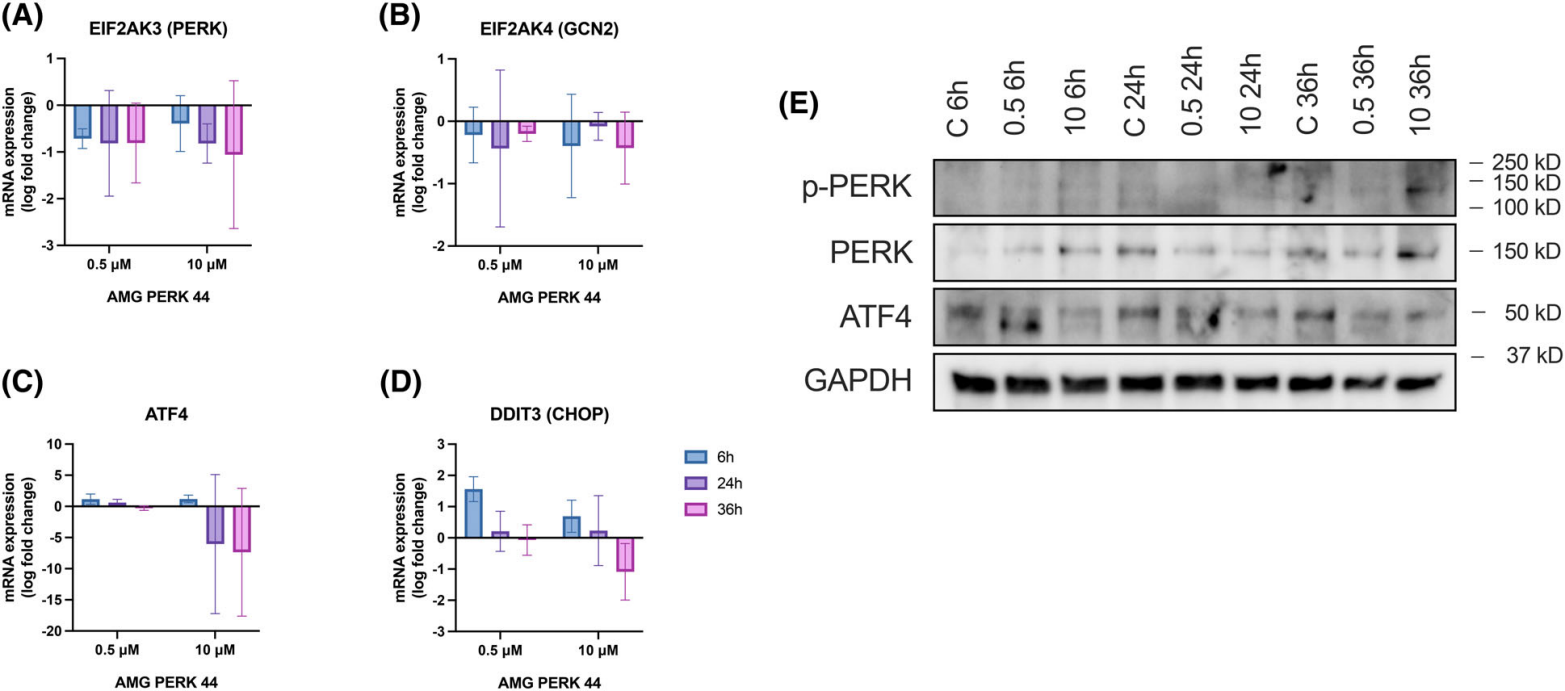
Effect of AMG PERK 44 treatment on UPR‐related gene expression *in vitro*. mRNA expression levels of EIF2AK3 (PERK) (A), EIF2AK4 (GCN2) (B), ATF4 (C), and DDIT3 (CHOP) (D) were quantified by qRT–PCR in cell lysates treated with 0.5 μm or 10 μm AMG PERK 44 for 6, 24, or 36 h. Data are presented as log fold change relative to untreated control (set to 0). Relative gene expression was analyzed using two‐way ANOVA followed by Tukey's multiple comparisons test. (E) Western blot analysis of phosphorylated PERK, PERK, and ATF4 in cell lysates treated with 0.5 μm or 10 μm AMG PERK 44 for 6, 24, or 36 h. GAPDH was used as a loading control. Bars show mean ± SD.

To investigate the unexpected increase in *Eif2ak3* transcription observed *in vivo* and to verify the activity of AMG PERK 44 *in vitro*, we performed acute (6 h) and prolonged (24–36 h) treatments of cultured cells using low (0.5 μm) and high (10 μm) concentrations of the inhibitor. Across all conditions, *Eif2ak3* mRNA levels showed a consistent modest decrease relative to control, with similar effects at both concentrations and across time points (Fig. 6A). Expression of *Eif2ak2* (GCN2) remained largely unchanged (Fig. 6B). *Atf4* mRNA levels were stable at 0.5 μm, whereas 10 μm treatment resulted in a transient increase at 6 h followed by a reduction at later time points (Fig. 6C). *Ddit3* (*Chop*) expression increased transiently at 6 h under both concentrations and decreased below baseline by 36 h at 10 μm (Fig. 6D). At the protein level, western blot detection of PERK, p‐PERK, and ATF4 resulted in weak but detectable bands. Under the high‐dose, prolonged condition (10 μm, 36 h), PERK protein levels appeared increased, and p‐PERK was detectable, although variability and low signal intensity limited quantitative interpretation (Fig. 6E).

**Fig. 7:**
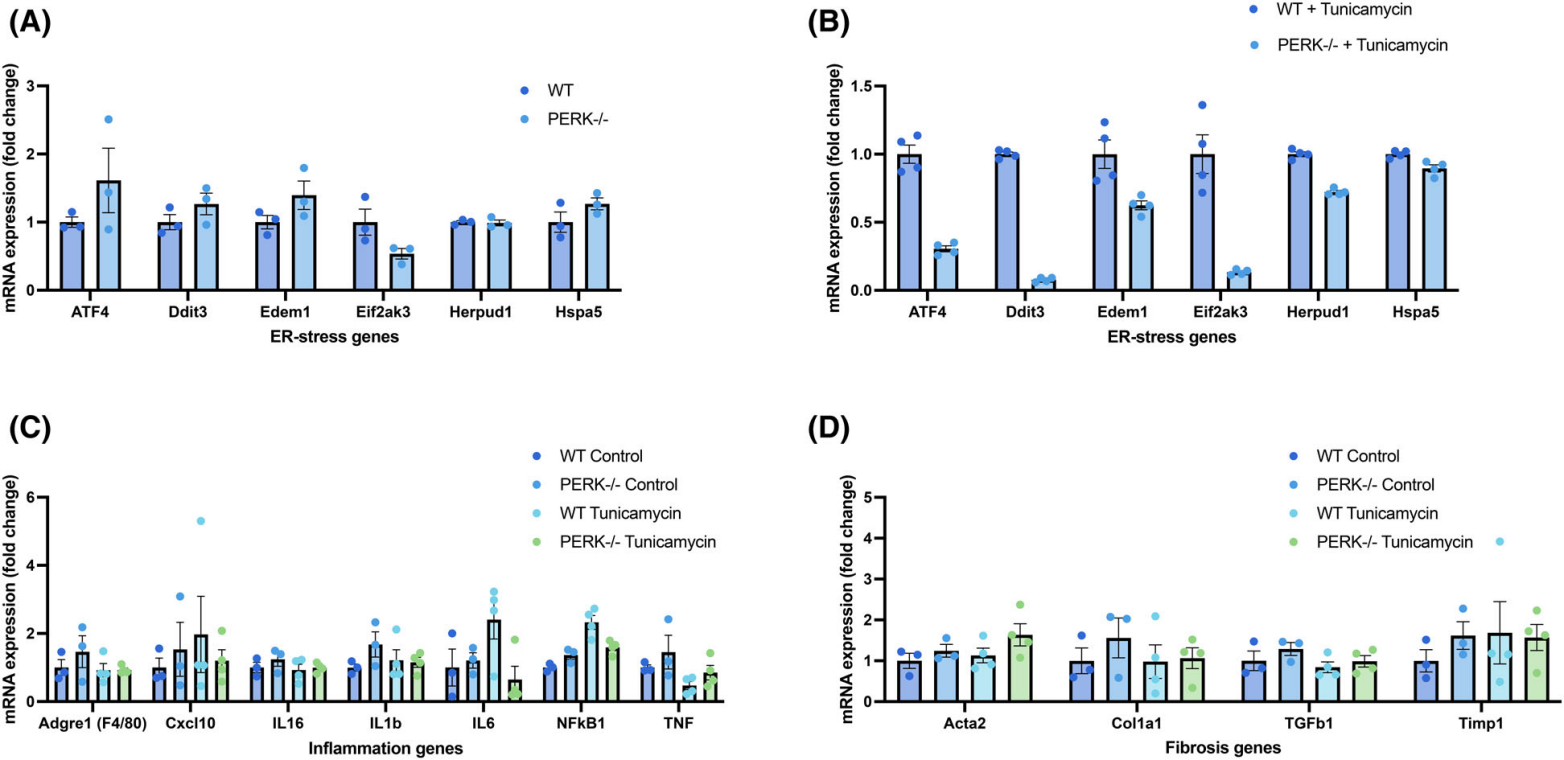
PERK deficiency alters ER stress, inflammatory, and fibrotic gene expression in response to tunicamycin. (A,B) Micro‐array analysis of endoplasmic reticulum (ER) stress‐related genes (ATF4, Ddit3, Edem1, Eif2ak3, Herpud1, Hspa5) in primary hepatocytes from wild‐type (WT) and PERK^−^/^−^ mice under basal conditions (A) or after tunicamycin treatment (B) to induce ER stress. Data are expressed as fold change relative to untreated WT controls. (C) mRNA expression of inflammatory genes (Adgre1/F4/80, Cxcl10, IL16, IL1b, IL6, NFκB1, TNF) in hepatocytes from WT and PERK^−^/^−^ mice, either untreated or treated with tunicamycin. (D) mRNA expression of fibrosis‐related genes (Acta2, Col1a1, TGFb1, Timp1) in the same experimental conditions. Background correction, normalization, and summarization were performed using the Robust Multi‐array Average (RMA) method. Each dot represents an individual sample. Bars show mean ± SD.

To further define how PERK inhibition influences the UPR and its potential effect on inflammatory and fibrotic signaling, we analyzed a publicly available transcriptomic dataset (GSE29929) including liver samples from wild‐type (WT) and liver‐specific PERK knockout (PERK^−/−^) mice, with or without treatment with the ER stress inducer tunicamycin [24]. PERK^−/−^ livers displayed a modest but consistent increase in the expression of several UPR genes, including *Atf4*, *Edem1*, *Hspa5* (BiP), and *Ddit3* (CHOP), compared to WT controls (Fig. 7A). Although these differences did not reach statistical significance, the coordinated transcriptional upregulation of these UPR markers suggests a mild activation of compensatory ER stress signaling in PERK‐deficient livers under basal conditions. Interestingly, under tunicamycin‐induced ER stress, PERK^−/−^ mice exhibited a suppression of UPR gene expression compared to WT counterparts, with nearly all canonical ER stress markers being markedly reduced in the absence of PERK (Fig. 7B). Together, these data suggest that while PERK loss can trigger low‐level compensatory ER stress responses in homeostatic liver tissue, it impairs the full transcriptional activation of the UPR under acute stress conditions.

To determine whether PERK influences inflammatory signaling pathways in the liver, we analyzed the expression of inflammation‐related genes. In control PERK^−/−^ livers, several genes (*Il6*, *Cxcl10*, *Adgre1*) trended toward higher mRNA expression compared to WT livers, similar to what we observed after pharmacological treatment with AMG PERK 44 (Fig. 7C). However, under ER stress conditions, tunicamycin‐treated PERK^−/−^ livers showed a broad reduction in inflammatory gene expression relative to WT‐treated samples. Notably, expression of *Tnf*, *Il6*, and *Il1b* was markedly suppressed in the absence of PERK signaling during ER stress, suggesting that PERK contributes to the transcriptional activation of inflammatory mediators in response to acute stress. However, no significant or physiologically relevant differences were observed in fibrotic markers between any of the groups (Fig. 7D).

### Inhibiting PERK did not enhance the effect of idarubicin

**Fig. 8:**
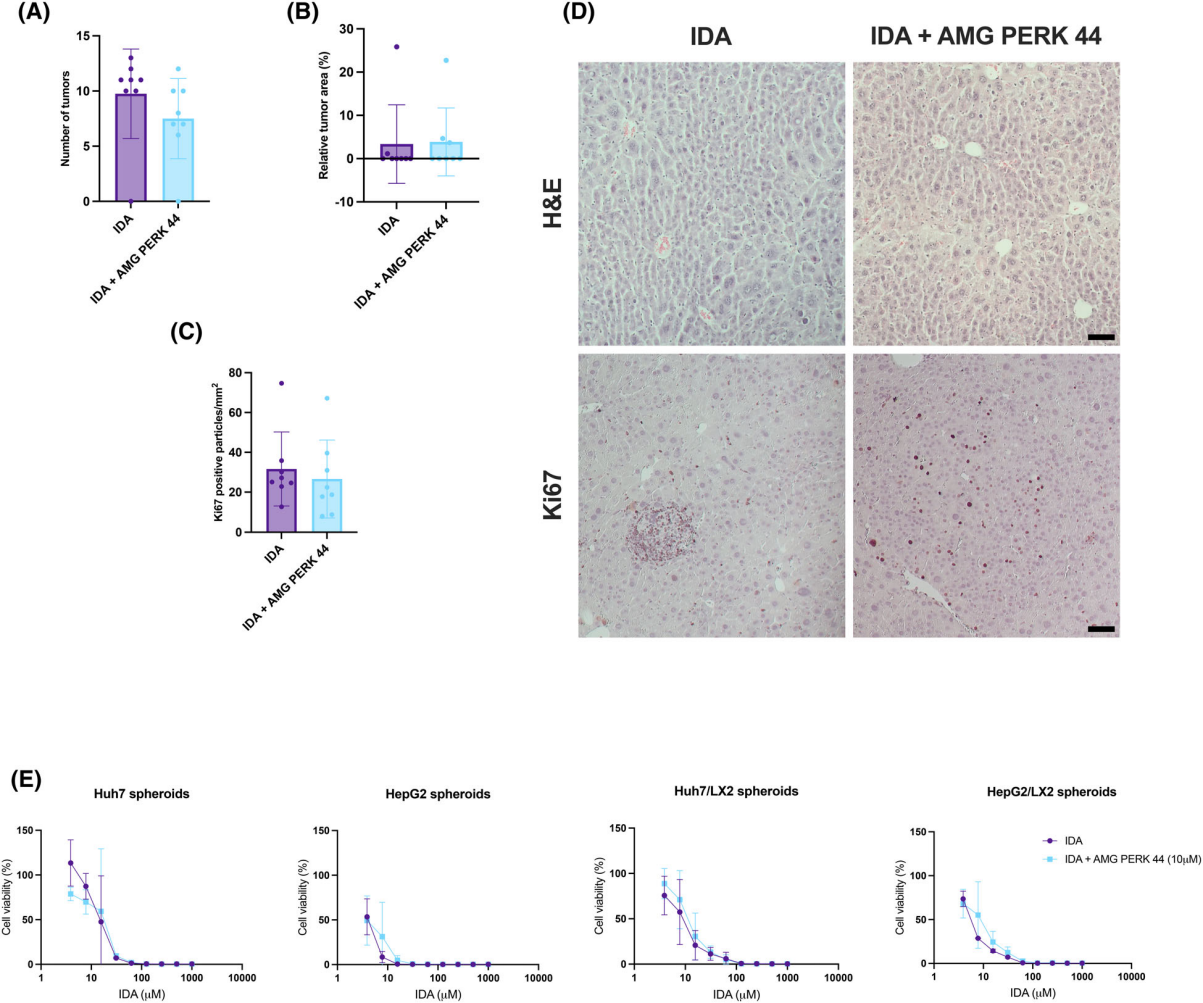
Inhibiting PERK did not enhance the effect of idarubicin. Quantification of tumor number (A), relative tumor area (B), and Ki67‐positive cells (C) in livers from mice treated with IDA alone or in combination with the PERK inhibitor AMG PERK 44. Bars represent mean ± SD. Statistical significance was determined using one‐way ANOVA followed by Tukey's multiple comparisons test. Each dot corresponds to one mouse. *N* = 7–8 mice. (D) Representative images of H&E and Ki67 immunostaining. (E) Dose–response curves showing cell viability (%) after treatment with increasing concentrations of IDA (purple) or IDA + AMG PERK 44 (10 μm) (blue) in 3D spheroid models of Huh7, HepG2, Huh7/LX2, and HepG2/LX2. Bars represent mean ± SD.

To assess the impact of AMG PERK 44 on the efficiency of IDA, a commonly used anthracycline in the context of transarterial chemoembolization in HCC treatment [17, 28], we compared the effect of IDA (0.4 μg/g body weight) alone to the combined effect of IDA + AMG PERK 44 (10 μg/g body weight) in mice with DEN‐induced HCC. To determine the impact of PERK inhibition on tumor development and growth, we quantified the number and size of liver tumors, as well as cellular proliferation rates across the DEN+IDA and DEN+IDA + AMG PERK 44 groups (Fig. 8A–D). In our animal model, AMG PERK 44 in combination with IDA did not reduce the number of tumors, tumor area, or overall proliferation at the doses used in this study. Moreover, we did not observe any enhancement of the cytotoxic effect of IDA when used in combination with AMG PERK 44 across multiple *in vitro* spheroid models and a broad concentration range (Fig. 8E and Table 1).

**Table 1:** IC50 values from cytotoxicity assays.

Culture	IC50 IDA	IC50 IDA + AMG PERK 44
Huh7 spheroids	14,38	13,76
HepG2 spheroids	2512	3314
uh7/LX2 spheroids	7908	10,97
HepG2/LX2 spheroids	5051	7527

### Inhibiting PERK did not cause off‐target effects

**Fig. 9:**
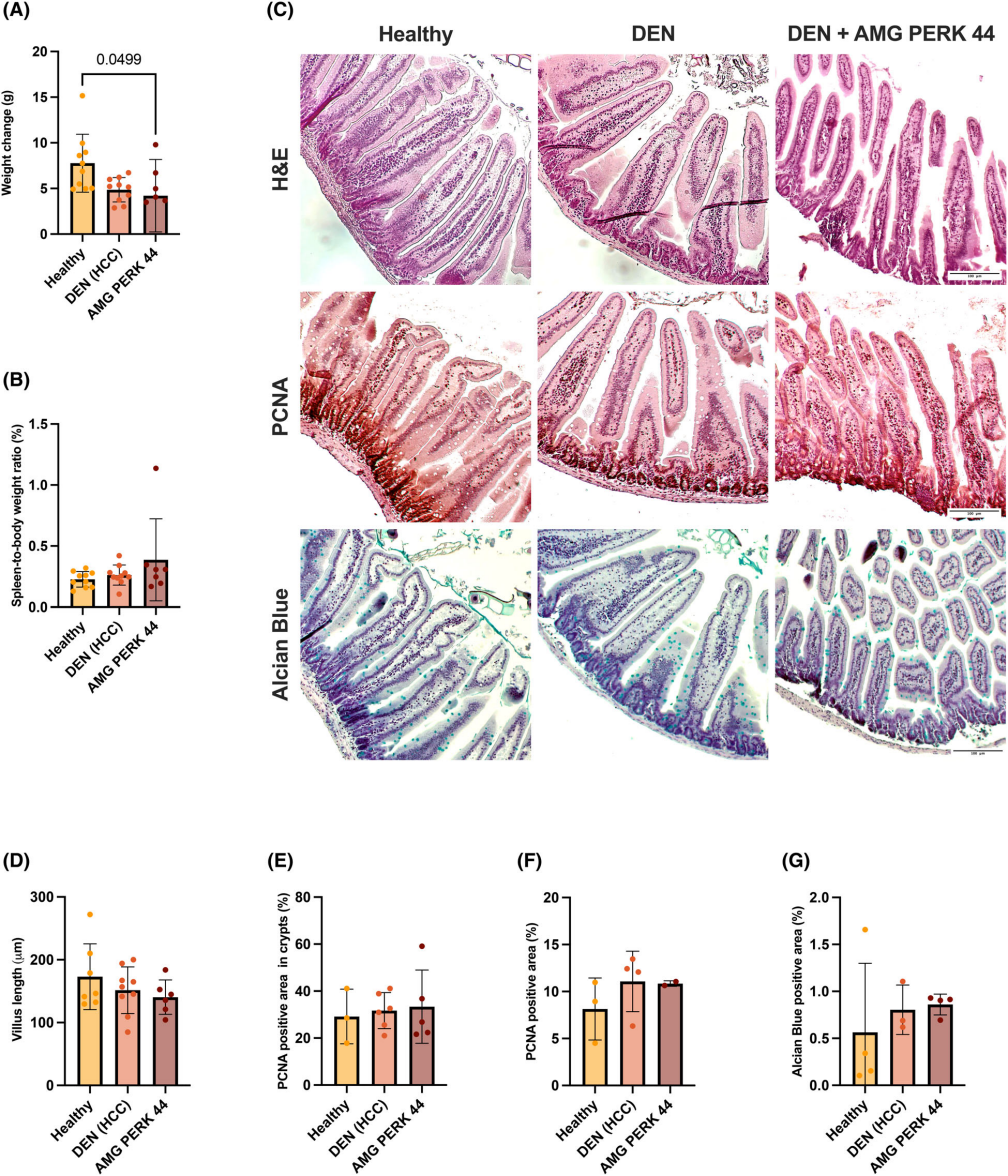
AMG PERK 44 did not cause side effects. Body weight change (A) and relative spleen‐to‐body weight ratio (B) in healthy, DEN (HCC), and AMG PERK 44–treated mice. (C) Representative images of H&E, PCNA, and Alcian Blue staining of small intestinal sections from each group. Quantification of villus length (μm) (D), PCNA‐positive area and crypt localization (%) (E,F), and Alcian Blue–positive area (%) (G). Bars represent mean ± SD. Statistical significance was determined using one‐way ANOVA followed by Tukey's multiple comparisons test. Each dot corresponds to one mouse. *N* = 2–10 mice. Scale bars represent 100 μm.

Mice with DEN‐induced HCC treated with biweekly i.p. injections of AMG PERK 44 displayed a significant reduction in total body weight compared to healthy control animals (Fig. 9A). However, there was no significant difference in body weight between DEN and DEN+AMG PERK 44 groups. The spleen‐to‐bodyweight ratio, a surrogate marker of systemic inflammation and disease burden, did not differ significantly among the study groups (Fig. 9B). This suggests that AMG PERK 44 treatment did not substantially alter splenic enlargement or the overall inflammatory status of the animals. These data indicate that while AMG PERK 44 may influence body weight, it does not exacerbate or alleviate systemic inflammation as measured by spleen index in this model.

PERK activity may also affect highly secretory and regenerative tissues such as the intestinal epithelium, where it supports epithelial renewal and goblet cell function [29]. Thus, we investigated gastrointestinal (GI) integrity in the jejunum in the different treatment groups. H&E staining of jejunal sections revealed no significant morphological alterations among experimental groups (Fig. 9C) [22, 23]. Villus length was similar in AMG PERK 44 relative to DEN (HCC) controls, indicating that overall intestinal architecture was preserved (Fig. 9C,D). Assessment of epithelial proliferation by PCNA staining showed a similar pattern across all groups (Fig. 9C, E and F). The number and distribution of PCNA‐positive cells were consistent with those observed in HCC controls, suggesting that epithelial renewal capacity remained stable without any compensatory proliferative response occurrence. Evaluation of mucin production using Alcian Blue staining demonstrated uniform goblet cell distribution and mucin content across all groups (Fig. 9C and G). The absence of detectable changes indicates that goblet cell function and mucosal barrier integrity and functions were maintained under all experimental *in vivo* conditions.

## Discussion

This study indicates that the PERK inhibitor AMG PERK 44 slightly affected tumor growth, yielding smaller tumors with fewer proliferative (e.g., Ki‐67^+^) cells, but did not reduce the number of tumors. This suggests that while PERK signaling is important for sustaining tumor expansion, it may not be essential for initial tumor emergence or seeding. This finding is in line with earlier studies showing that pharmacologic PERK blockade may inhibit tumor cell proliferation and xenograft growth *in vivo* [30]. Consistently, PERK inhibition in mice has been shown to reduce tumor growth, an effect that likely requires an intact immune system [31]. Thus, our observation that AMG PERK 44 affects tumor size (diminishing their proliferative cell fraction) without reducing tumor count might reflect PERK's role in enabling tumor cells to cope with stress and grow, rather than in the initial steps of tumor formation. Once tumors are established, PERK‐driven adaptive stress responses (e.g., limiting protein synthesis and oxidative damage) are known to promote cancer cell survival and proliferation [32]. By disabling this pathway, AMG PERK 44 likely pushes tumor cells past an ‘adaptive capacity’, curbing their growth. However, if microscopic tumors have already formed (or new lesions are continuously initiated by carcinogenic stimuli), PERK inhibition alone may not eliminate those nascent foci, explaining the unchanged tumor count. This distinction between tumor initiation vs. progression has been discussed before: downstream inhibition of PERK signaling, as well as early (and often unspecific) PERK inhibitors (like GSK2606414/GSK2656157) did not always prevent tumor growth, even though they affected pro‐invasive and metastatic functions at advanced stages [33]. AMG PERK 44's impact on tumor composition (size and proliferative index) aligns with the idea that PERK primarily confers a growth advantage to tumor cells under stress, rather than acting as an absolute requirement for tumorigenesis. From a therapeutic perspective, these findings are interesting, as targeting PERK can constrain tumor progression; but needs to be taken with caution. Even with effective PERK inhibition, residual dormant tumor cells or new tumors could persist. Combining PERK inhibitors with other treatments (e.g., cytotoxic or immune therapies) might be necessary to eliminate all tumorigenic cells, as hinted by studies where PERK blockade synergized with checkpoint inhibitors to produce stronger antitumor effects [19] and IRE1a inhibitors that enhanced the effect of doxorubicin [34]. However, our data did not support that combining AMG PERK 44 with IDA had any additive or synergistic effect, compared to the treatments alone. This could potentially be attributed to the fact that anthracyclines induce ER stress and ferroptosis in cancer cells, which contributes to their anticancer effect [17, 35]. Blocking the PERK pathway might have therefore interfered with these mechanisms of action. In summary, our data underscore the need to tackle tumor initiation or persistence via complementary mechanisms since PERK inhibition alone does not reduce tumor growth in an advanced biorelevant HCC model.

In our DEN model, AMG PERK 44 treatment was not associated with a reduction in collagen deposition nor fibrosis in the tumor‐bearing livers. While *α*‐SMA expression was significantly upregulated in the peritumoral areas (suggesting activation of hepatic stellate cells), PERK inhibition resulted in reduced *α*‐SMA expression. Interestingly, fibrotic mediators such as CTGF and TGF‐*β* were upregulated in the tumor regions of AMG PERK 44‐treated livers, indicating that PERK inhibition may enhance profibrotic signaling within the tumor microenvironment while decreasing HSC activation [36, 37, 38]. CTGF has been described to be transcriptionally activated by ATF6 [39] and it is possible that, because PERK activity is inhibited by AMG PERK 44 and the ER response remains active, another branch of the UPR (e.g., ATF6) compensates and drives CTGF expression. This could explain the selective increase of CTGF in tumor regions of our DEN model. Moreover, PERK is an ER stress sensor, and chronic ER stress has been implicated in promoting fibrosis in multiple organs via several mechanisms: prolonged UPR signaling (particularly the pro‐apoptotic factor CHOP) can drive apoptotic cell loss and subsequent inflammatory responses that activate fibrogenesis, and UPR mediators can crosstalk with profibrotic pathways like TGF‐*β* in fibroblasts and stellate cells [10] as well as by affecting macrophage polarization and function [40, 41, 42].

In addition to the elevated profibrotic markers, pro‐inflammatory markers such as IL‐6, IL‐1B, and TNF‐*α* showed higher expression in tumor regions, yet these changes were not significant. Notably, PERK inhibition further increased the expression of IL‐6 and TNF‐*α*, indicating enhanced inflammatory signaling within the tumor. This inflammatory signature was accompanied by increased macrophage infiltration, as shown by the marker F4/80, which was also significantly elevated upon PERK inhibition. Interestingly, immunofluorescent analysis of F4/80 protein in tissue revealed that macrophages did not appear to infiltrate the tumor to a greater extent. This hints that PERK inhibition may influence macrophage transcriptional activity or phenotype rather than cell number or infiltration. It could be that PERK affects macrophage polarization, influencing the balance between pro‐inflammatory and immunosuppressive phenotypes within the immune response [40, 42].

In our study, treatment with AMG PERK 44 was associated with increased expression of several UPR‐related genes in tumor tissue, which may appear counterintuitive given that PERK is one of the ER stress sensors we aimed to inhibit. However, this finding should be interpreted cautiously. The UPR is comprised of three interconnected branches (PERK, IRE1, and ATF6), and inhibition of one arm can disrupt adaptive buffering mechanisms, inadvertently promoting the accumulation of misfolded proteins and enhancing activation of the remaining sensors. Normally, PERK reduces protein synthesis (via eIF2*α* phosphorylation) and upregulates stress‐alleviating factors like ATF4; its inhibition can remove this translational brake, increasing the protein load entering the ER and exacerbating stress, particularly in a hostile tumor microenvironment (e.g., hypoxia and/or nutrient deprivation) [5]. Because perturbations in ER homeostasis impair protein folding, they elicit a compensatory UPR characterized by broad translational and transcriptional adjustments aimed at expanding ER processing capacity and mitigating injury. The result can be a reactive increase in chaperones and stress signals. In fact, UPR signaling is a delicate balance [43], evidence suggests that PERK activity is required for the proper timing of ATF6 activation under stress [24]. When PERK is pharmacologically inhibited, the coordination of UPR signaling is disrupted, potentially causing ATF6 and IRE1 to hyperactivate in an attempt to compensate. Our findings echo the scenario reported by Mandula et al. and others in 2022, where blocking PERK led to severe ER enlargement and stress in tumors in melanoma‐bearing mice, ultimately inducing a form of cell death (paraptosis) accompanied by robust CHOP expression and type I interferon signaling [31]. Interestingly, our analysis of liver‐specific PERK knockout mice in both the presence and absence of tunicamycin‐induced stress provides further nuance to this paradox. In homeostatic liver tissue, genetic deletion of PERK led to a mild but consistent upregulation of several canonical UPR genes, including *Atf4*, *Hspa5*, *Edem1*, and *Ddit3*, despite complete loss of *Eif2ak3* expression. This suggests that even under basal conditions, PERK deficiency may induce low‐level compensatory stress signaling, likely via crosstalk with other UPR branches. However, under acute ER stress induced by tunicamycin, PERK knockout profoundly decreased the transcriptional activation of these same genes, indicating that PERK is required for a full UPR response under stress. In our mouse model, we observe a significant increase in CHOP, EIF2AK3, and ERO1b mRNA expression in tumor tissue after treatment with AMG PERK 44. Our *in vitro* experiments showed modest and variable changes in UPR‐related gene expression after acute and prolonged AMG PERK 44 exposure, while protein‐level readouts (PERK, p‐PERK, ATF4) were weak and did not reveal clear treatment‐dependent effects. Together with the absence of *in vivo* liver drug concentration measurements, these limitations make it difficult to draw firm conclusions about the degree of PERK pathway modulation achieved in the tumor microenvironment.

AMG PERK 44 has been reported to inhibit GCN2 at higher micromolar concentrations in some contexts [13]. Although our data did not show major changes in Eif2ak2 expression, we cannot exclude potential contributions from other cellular stress sensors, particularly in conditions where ER stress is already elevated. This possibility is especially relevant for the higher concentration used in spheroid assays. The increases in UPR‐associated transcripts observed in tumor tissue likely reflect the context and time‐dependent nature of ER stress signaling. Given the modest *in vitro* effects, the weak *in vivo* protein signals, and the lack of pharmacokinetic information, these findings should be interpreted conservatively. Notably, phosphorylation of eIF2*α* (p‐eIF2*α*), a key downstream readout of PERK activity, was not assessed *in vivo*, which limits conclusions regarding PERK pathway inhibition at the functional level. In addition, GCN2 kinase activity was not evaluated at the protein level. Additional experiments assessing p‐eIF2*α*, GCN2 activity, or broader UPR outputs will be needed to fully determine how AMG PERK 44 affects stress response pathways in this setting.

Across all treatment groups, intestinal mucosal villus height, epithelial proliferation, and mucin production remained unchanged. The absence of morphological or proliferative changes suggests that AMG PERK 44 did not disrupt epithelial turnover or compromise intestinal mucosal integrity, consistent with reports that PERK inhibition has minimal impact in nonstressed tissue due to compensatory UPR pathways [44]. Overall, these findings demonstrate that AMG PERK 44 is well tolerated at the intestinal level and did not cause any significant intestinal side effects [45, 46].

In conclusion, our study provides experimental results on using PERK inhibition as a strategy to restrain tumor growth and proliferation, but perhaps more importantly, by affecting the tumor microenvironment. The paradox of elevated ER stress markers is explained by known UPR dynamics and cell‐death feedback, aligning our data with the concept of inducing lethal stress in cancer cells. Each of these points strengthens the interpretation of AMG PERK 44's impact and suggests avenues for further research, such as combining PERK inhibitors with immunotherapy or fibrosis inhibitors.

## Conflict of interest

The authors declare no conflicts of interest.

## Author contributions

FH conceived and designed the research, drafted the first manuscript and secured research funding. ALC, MK and NA performed experiments. FH, ALC, MK and NA and analyzed data. FH, ALC, MK, NA, MS and HL interpreted results of experiments. All authors edited and revised the manuscript. All authors approved the final version of the manuscript and agree to be accountable for all aspects of the work in ensuring that questions related to the accuracy or integrity of any part of the work are appropriately investigated and resolved. All persons designated as authors qualify for authorship, and all those who qualify for authorship are listed.

## Use of artificial intelligence

AI tools (ChatGPT, OpenAI) were used for language editing during manuscript preparation, in accordance with FEBS Press guidelines. The authors reviewed all content and take full responsibility for the manuscript.

## Data Availability

Data are available upon request. Please note that the ‘Healthy’ and ‘DEN’ and ‘DEN + idarubicin’ control groups have also been included in Lerma‐Clavero A *et al*. [17], as the animal experiments for both publications were conducted simultaneously, with different treatment arms but shared controls. This dual use of control groups adheres to the 3R principle by minimizing animal use.
